# Two Active Compounds from *Caesalpinia sappan* L. in Combination with Cisplatin Synergistically Induce Apoptosis and Cell Cycle Arrest on WiDr Cells

**DOI:** 10.15171/apb.2017.045

**Published:** 2017-09-25

**Authors:** Sri Handayani, Ratna Asmah Susidarti, Riris Istighfari Jenie, Edy Meiyanto

**Affiliations:** ^1^Research Center for Chemistry, Indonesian Institute of Sciences (LIPI), Serpong, Indonesia.; ^2^Cancer Chemoprevention Research Center, Faculty of Pharmacy,Universitas Gadjah Mada, Yogyakarta, Indonesia.

**Keywords:** Brazilin, Brazilein, Caesalpinia sappan, L., Cisplatin, Synergistic effect, WiDr cells

## Abstract

***Purpose:*** The aim of this study is to observe the synergistic effect of two active compounds of secang, brazilin and brazilein, combined with cisplatin on WiDr colon cancer cells.

***Methods:*** Cytotoxic activities of brazilin (Bi) and brazilein (Be) in single and in combination with cisplatin (Cisp) were examined by MTT assay. Synergistic effect was analyzed by combination index (CI) parameter. Apoptosis and cell cycle profiles were observed by using flow cytometry.

***Results:*** The result of MTT assay showed that IC_50_ value of brazilin and brazilein on WiDr cancer cells were 41 µM and 52 µM respectively. The combination of ½ IC50 of Bi-Cisp reduced cells viability up to 64% and showed synergistic effect with CI value less than 1 (CI = 0.8). The combinations of ½ IC_50_ of Be-Cisp also reduced cells viability up to 78% and showed synergistic effect (CI=0.65). Combination of Bi-Cisp and Be-Cisp induced apoptosis higher than the single treatments. Further analysis on the cell cycle progression showed that single treatment of ½ IC_50_ of Be and Bi induced S-phase and G2/M-phase accumulation, while combination of Be-Cisp and Bi-Cisp enhanced S-phase accumulation.

***Conclusion:*** Both combination of Bi-Cisp and Be-Cisp showed synergistic effect on WiDr cells through induction of apoptosis and halted the cell cycle progression, thus, WiDr cells growth were significantly reduced.

## Introduction

Some of colorectal cancer (CRC) cases are associated with poor survival because of *p53* mutation.^1^ The *p53* gene is a tumor suppressor and the key regulator of DNA damage responses. This gene plays a role on tumor suppression processes including cell cycle arrest and apoptosis.^2^ Mutation in *p53* gene leading to the loss of wild-type *p53* activity is frequently detected in many different tumor types.^3^ Enhancing of invasiveness, attenuating of apoptosis and increasing of genomic instability are generally occurred after mutation of *p53.*^4^ The *p53*-mutant cancer may not be treated with the same agent as *p53* wild type cancer.

Surgery, chemotherapeutic drugs and radiation therapy are some of the cancer therapies that frequently be used to treat colon cancer patient.^5^ Cisplatin and its derivatives are the effective DNA-damaging anti-tumor agent for various human cancers, including colon cancer.^6^ The p53 protein is stabilized and its level increases in response to various DNA damaging agents, including cisplatin. However, side effects including resistance to cisplatin arise in *p53-*mutant cancer cells.^7^ Thus, we need to investigate potential anticancer compounds that work on p53-independent pathway. Currently, combination of chemotherapy regiments based on platinum-derived compounds with other drugs (co-chemotherapeutic agent) constitute the pharmacological therapy of choice for the treatment of colon cancer. The *p53*-mutant colon cancer may need to be treated with combination therapy.

The major compounds of *Caesalpinia sappan* L. (Caesalpiniaceae) i.e. brazilin and brazilein have been reported to have activities as antiinflammation, antioxidant, hepatoprotective and antiviral.^8-11^ Brazilin and brazilein induce apoptosis and inhibit cell growth on several cancer cells.^12-14^ Brazilein suppresses cancer cells migration and invasion.^15^ Thus, brazilin and brazilein have potential to be developed as co-chemotherapeutic agent. Nevertheless, synergistic effect of combination of brazilin and brazilein with cisplatin against *p53-*mutant WiDr cancer cells has never been reported. In this study, we observed the synergistic cytotoxic effect of combination of brazilin and brazilein with cisplatin on WiDr cancer cells.

## Materials and Methods

### Chemicals

Brazilin and brazilein were isolated from *Caesalpinia sappan,* L. using previously reported method.^9^ Cisplatin was purchased from Wako (Japan).

### Cell culture

WiDr cell line was obtained Faculty of Medicine, Universitas Gadjah Mada, Yogyakarta.

### Cytotoxic assay

The 1×104 cells/well were seeded in 96-well plate. Cells were treated with various concentrations of brazilin, brazilein, and cisplatin in single or in combination then incubated in 37°C, 5% CO_2_ for 24 h. Cells were then washed with PBS (Sigma), were treated with 100 µL culture medium containing 0.5 mg/mL MTT (Sigma) and incubated in 37°C, 5% CO_2_ for 4 h. The reaction was stopped by adding to the cells with SDS reagent (10% sodium dodecyl sulphate (Merck) in HCl 0.01M (Merck)) then was incubated overnight in room temperature. The absorbance was measured with microplate reader (Bio-Rad) at λ 595 nm.

### Cell cycle distribution

Propidium iodide (PI) staining kit (Becton Dickinson) was used to analyze DNA content. 5×104 cells/well were seeded in 24-well plate. Cells were treated with various concentrations of samples in single or in combination then incubated in 37°C, 5% CO_2_ for 24 h. Cells were harvested and washed in PBS, fixed with 70% ethanol, labeled with PI (2 µg/mL), and incubated in room temperature, protected from light, for 10 min. The DNA content was analyzed using flow cytometry (Becton Dickinson) and followed by flowing software (version 2.5.1) and Excel MS Office 2013.

### Apoptosis detection

Apoptotic cells population was determined using PI-Annexin V assay (Annexin V-FITC Apoptosis Detection Kit Roche). 5×10^4^ cells/well were seeded in 24-well plate. Cells were treated with various concentrations of samples either single or in combination for 24 h. Cells were harvested, added with 1× binding buffer, labeled with PI-Annexin V, and incubated at room temperature in the dark for 5 min. The cells suspension was analyzed using a flow cytometry (Becton Dickinson) and followed by flowing software (version 2.5.1) and Excel MS Office 2013.

### Statistical analysis

Statistical analysis was performed using Student t-test (Microsoft Excel 2013). *P*-values less than 0.05 were considered significant.

Effects of combinations on growth inhibition were analyzed using the Combination index (CI) equation developed by Reynolds and Maurer.^16^

## Results and Discussion

### Cytotoxic effect of brazilin and brazilein on WiDr cells.

**Figure 1:**
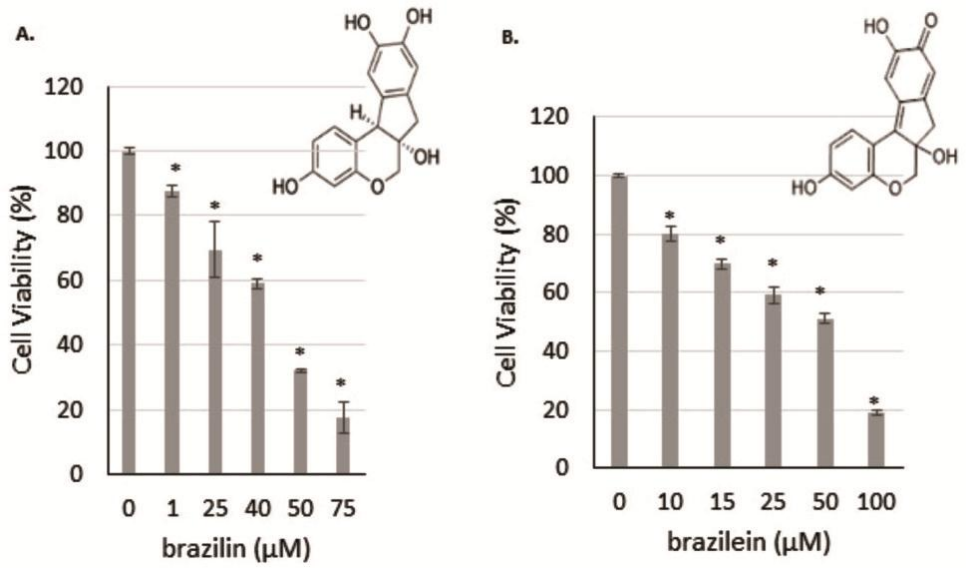
Cytotoxic effect of brazilin and brazilein on WiDr cells. A. brazilin (1-75 µM), B. brazilein (10-100 µM). Cells were treated with various concentrations of samples for 24 h before assessed by MTT assay. Data was collected from three replications (P<0.05).

First, we observed the cytotoxic activity to find out the potency of brazilin and brazilein in inhibiting of WiDr cells proliferation. The cytotoxic activity was performed by using MTT assay. The results showed that brazilin and brazilein inhibited WiDr cells growth in a dose-dependent manner with IC_50_ value were 41 µM and 52 µM respectively (Figure 1), while the IC_50_ value of cisplatin was 15 µM (data not shown).

### Synergistic effect of combination of Bi-Cisp and Be-Cisp on WiDr cells growth

**Figure 2:**
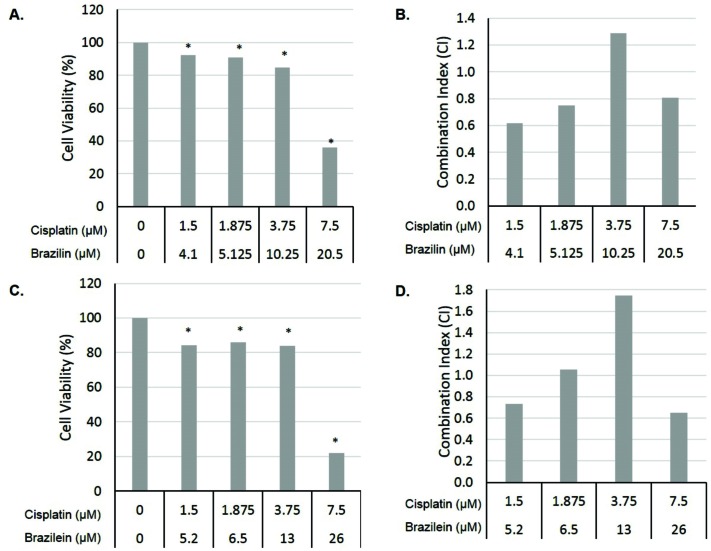
Combination of ½ IC_50_ of Bi-Cisp (20.5 µM- 7.5 µM) and Be-Cisp (26 µM- 7.5 µM) synergistically inhibited WiDr cells growth. A. Effect of combination of Bi-Cisp on WiDr cells growth. B. Combination Index (CI) of Bi-Cisp on WiDr cells. C. Combination of Be-Cisp on WiDr cells growth. D. CI of Be-Cisp on WiDr cells. Cells were treated with samples for 24 h before assessed by MTT assay. Data was collected from three replications (P<0.05).

Discovery of agents that reduce resistance of chemotherapeutic agent is urgently needed and become one main target of today research. We investigated the effect of brazilin and brazilein in combination with cisplatin, a standard chemotherapeutic agent, on WiDr colon cancer cells. As shown in Figure 2, the combination of 1/10, ⅛, ¼ and ½ IC_50_ of either Bi-Cisp or Be-Cisp showed synergistic effect on inhibition of WiDr cells growth (CI<1). Moreover, the combination of ½ IC_50_ of Bi-Cisp inhibited cell viability up to 64%. These data indicate that both compounds are potential to be developed as co-chemotherapeutic agent on colon cancer cells. To understand the mechanism underlies the synergistic effect, we observed the effect of the combinations on cell cycle modulation and on the induction of apoptosis.

### Cell cycle modulation of brazilin and brazilein in combination with cisplatin

**Figure 3:**
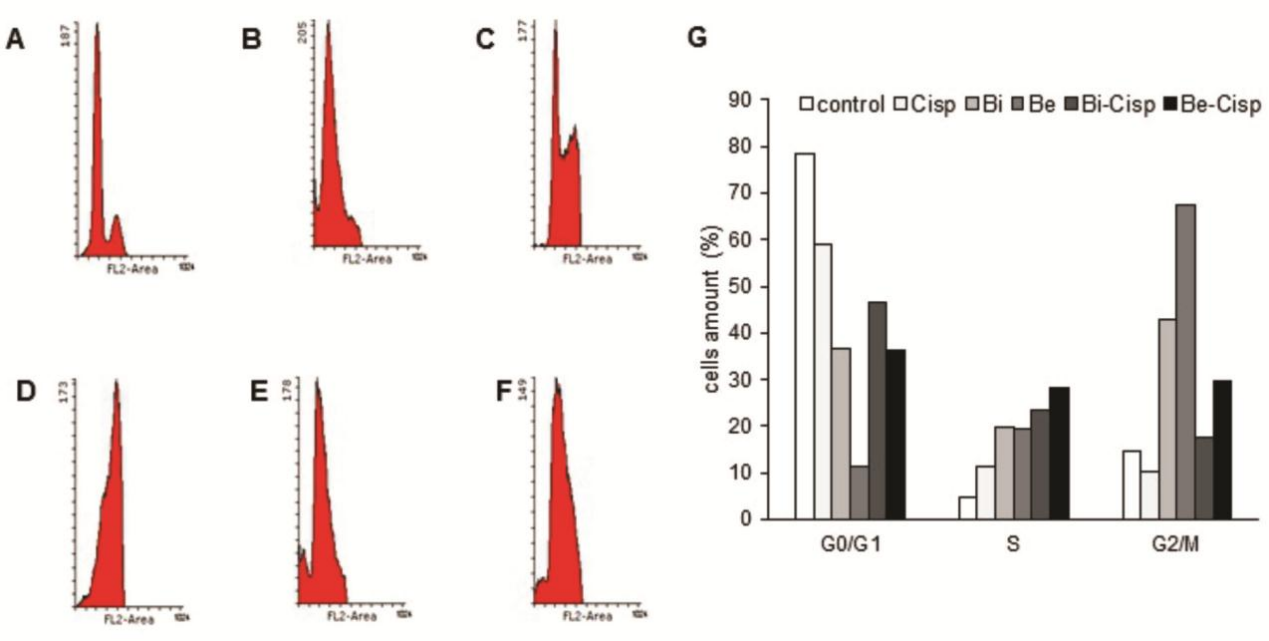
Combination of ½ IC_50_ of Bi-Cisp and Be-Cisp induced S-phase accumulation on WiDr cells. A. Control. B. Cisplatin (7.5 µM). C. Brazilin (20.5 µM). D. Brazilein (26 µM). E. Combination of Bi-Cisp (20.5 µM -7.5 µM). F. Combination of Be-Cisp (26 µM -7.5 µM). G. Cell cycle distribution of ½ IC_50_ of Bi and Be in single and combination with Cisp. Data was collected according to the description in Method.

We conducted cell cycle analysis to elucidate how brazilin, brazilein and their combination with cisplatin inhibited cells proliferation. The results of cell cycle analysis (Figure 3) showed that single treatment of either brazilin or brazilein caused S-phase and G2/M-phase accumulation. Treatment of ½ IC_50_ of brazilein (Figure 3D) induced higher G2/M accumulation compared to ½ IC_50_ of brazilin (Figure 3C). On the other hand, single treatment of ½ IC_50_ of cisplatin induced S-phase accumulation (Figure 3B). When brazilin or brazilein was combined with cisplatin, the combinations increased cells accumulation in S-phase compared to its single treatment, whereas both combination showed accumulation in G2/M-phase compared to control cells (Figure 3E-G).

### Apoptosis induction of brazilin and brazilein in combination with cisplatin.

**Figure 4:**
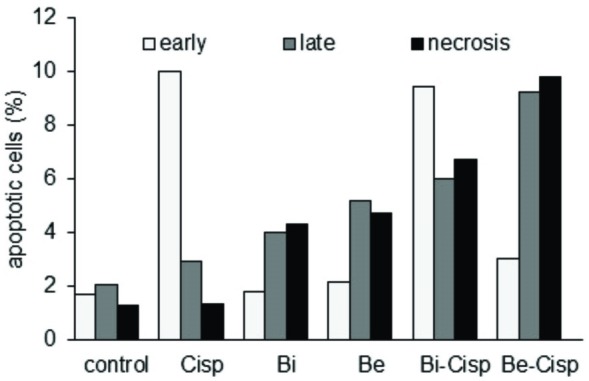
Combination of ½ IC_50_ of Bi-Cisp and Be-Cisp synergistically induced apoptosis on WiDr cells. Cells were treated with Bi, Be and Cisp (20.5 µM, 26 µM, and 7.5 µM respectively) as indicated in the graph for 24 h. Data was collected according to the description in Method.

Inhibition of proliferation may be caused by modulation in cell cycle and by the induction of apoptosis. To investigate the effect of brazilin, brazilein and their combination with cisplatin on apoptosis, we did FACS analysis on WiDr cells which were treated with samples and were stained with fluorescein isothiocyanate-conjugated annexin V and fluorescent dye propidium iodide (PI). Single treatment of cisplatin, brazilin and brazilein exhibited 14%, 10% and 12% of total apoptotic cells respectively. Either combination of brazilin-cisplatin or brazilein-cisplatin increased total apoptotic cells up to 22% compared to control or 9% compared to cisplatin alone (Figure 4). Combination of brazilein-cisplatin treatment showed highest accumulation in late apoptotic and necrosis, while combination of brazilin-cisplatin exhibited highest accumulation in early apoptotic cells following 24 hours of incubation. These results suggested that brazilin and brazilein increased the induction of apoptosis of cisplatin on WIDr cells.

Cisplatin is a platinum-based drug that is used for the treatment of a wide-variety of primary human cancers. However, the efficacy of cisplatin is often limited by the rise of drug resistance.^6,7^ Brazilin and brazilein are the active compounds from *Caesalpinia sappan* L. The structures of brazilin and brazilein are almost similar, whereas brazilein has C=O group and brazilin does not have C=O group in its structure.^9^ In this study, brazilin showed the better cytotoxic effect against WiDr cancer cells than brazilein (Figure 1). Nevertheless, both of compounds performed synergistic effect while were combined with cisplatin.

Cell cycle analysis showed that single treatment of either brazilin or brazilein induced S-phase and G2/M-phase accumulation, while single treatment of cisplatin only accumulated cells in S-phase (Figure 4). Interestingly, when brazilin and brazilein were given as combination with cisplatin, both compounds seemed to work effectively in two fields. Both brazilin and brazilein enhance S-phase accumulation of cell cycle up to 23% and 28% respectively compared to single treatment of cisplatin. The rest of the cells, which were lead to G2/M phase were also blocked by brazilin and brazilein (Figure 4). The mechanism underlies the S-phase and G2/M-phase arrest-induced by brazilin and brazilein needs further investigation. Previous study reported that brazilin induces G2/M arrest through inactivation of histone deacetylase and followed by the activation of CDK inhibitor p27.^14^ The G2/M accumulation on brazilein treatment on colon cancer cells may be related with the ability of brazilein to bind with COX2 receptor (data not shown). Brazilein may induce G2/M arrest through inhibition of COX2, which is followed with the activation of p21. The p21 inhibits the CDK1/cyclin B complex and maintains the G2 arrest.^17^ Since WiDr cell expresses *p53* mutant, p21 may be induced by p53-independent pathway.^18,19^ The S phase arrest-induced by the combination of brazilin-cisplatin and brazilein-cisplatin are possibly due to the action of cisplatin which as reported by Yuan et al,^20^ single treatment of cisplatin induces S-phase arrest via activation of CHK1 and CHK2 checkpoint kinase. The proteins induce cell death through DNA damage and cell cycle arrest during S phase. Hence, S-phase arrest induced by cisplatin through CHK1 activation on p53-mutant cells has been reported.^21^ Hence, we need to investigate that S-phase arrest induced by both of the combinations underwent apoptosis instead of DNA repair.

Although the total apoptosis event of single treatment of cisplatin, brazilin and brazilein were similar (Figure 2), the three compounds have different pattern. Cisplatin exhibited the highest distribution in early apoptosis. On the other hand, brazilin and brazilein exhibited the highest distribution in late apoptosis. Combination of both brazilin-cisplatin and brazilein-cisplatin enhanced induction of apoptosis compared to cisplatin single treatment. The results support our suggestion that S-phase accumulation induced by the combinations lead to apoptosis instead of DNA repair. Interestingly, even though the total apoptotic events of both of the combinations were similar, combination of brazilin-cisplatin exhibited the highest accumulation in the early apoptotic cells, while combination of brazilein-cisplatin treatment exhibited the highest accumulation in the late apoptotic cells and necrosis. This difference may be due to its different mechanism. Previous study reported that cisplatin induces apoptosis through FAS receptor pathway.^22^ FAS/FADD as well as TNF activate procaspase-8 to form caspase-8 and lead to increasing of proapoptotic protein Bax via intrinsic pathway.^23^ Caspase-8 also directly activated caspase-3 and 7 and lead to apoptosis via extrinsic pathway.^24^ Furthermore, brazilin induces apoptosis by increasing of cleavage caspase 3, cleavage caspase 7 and cleavage PARP,^12^ while brazilein directly inhibits anti apoptotic protein surviving.^25^ The *p53* is a transcription factor for pro-apoptotic protein, while *NFκB* is a transcription factor for anti-apoptotic protein such as Bcl-2 and surviving.^26^ Hsieh reported that brazilein inhibits activation of NFκB.^15^ On *p53*-mutated cells with loss most of pro-apoptotic protein expression, inhibition of NFκB activation which down-regulates anti-apoptotic protein and followed by apoptosis induction is an important mechanism to suppress cancer cells growth. Downregulation of of anti-apoptotic protein induces activation of caspase-9 and caspase-3 and followed with apoptosis phenomena.^25,27,28^ Those support our finding that the combination of brazilin-cisplatin and brazilein-cisplatin work synergistically to induce apoptosis on WiDr cells.

Combination of several therapies facilitates the blockade of several survival mechanisms in cancer cells and their microenvironment, achieving a synergistic therapeutic effect.^29^ On combination therapy, reducing of concentration without reducing the effect may be feasible. It overcomes side effects of chemotherapeutic agent. Brazilin and brazilein are potential to be developed as a co-chemotherapeutic agent with cisplatin for treating colon cancer cells. However, further investigation need to be done to elucidate the molecular mechanism of brazilin, brazilein and its combination with cisplatin in inducing apoptosis and modulating S-phase and G2/M arrest on WiDr colon cancer cells.

## Conclusion

Based on these results, we propose that brazilin and brazilein work synergistically with cisplatin in inhibiting colon cancer WiDr cells growth through a different target in cell cycle modulation and apoptosis induction.

## Acknowledgments

This work was supported by the grant of Penelitian Unggulan Perguruan Tinggi (PUPT) 2015 from Indonesian Ministry of Research and Technology and High Education.

## Ethical Issues

Not applicable.

## Conflict of Interest

The authors declare no conflict of interests.
